# Anthropometric indicators associated with high blood pressure in children living in urban and rural areas

**DOI:** 10.1590/1518-8345.2760-3150

**Published:** 2019-04-29

**Authors:** Gisele Nepomuceno de Andrade, Leonardo Ferreira Matoso, Jhon Wesley Bragança Miranda, Túlio Fonseca de Lima, Andréa Gazzinelli, Ed Wilson Vieira

**Affiliations:** 1 Universidade Federal de Minas Gerais, Escola de Enfermagem, Belo Horizonte, MG, Brasil; 2 Bolsista do Conselho Nacional de Desenvolvimento Científico e Tecnológico (CNPq), Brasil; 3 Bolsista da Coordenação de Aperfeiçoamento de Pessoal de Nível Superior (CAPES), Brasil; 4 Santa Casa de Belo Horizonte, Belo Horizonte, MG, Brasil; 5 Hospital Mater Dei, Belo Horizonte, MG, Brasil

**Keywords:** Child Health, Arterial Pressure, Body Mass Index, Waist Circumference, Anthropometry, Public Health, Saúde da Criança, Pressão Arterial, Índice de Massa Corporal, Circunferência da Cintura, Antropometria, Saúde Pública, Salud del Niño, Presión Arterial, Índice de Masa Corporal, Circunferencia de la Cintura, Antropometría, Salud Pública

## Abstract

**Objective:**

to evaluate anthropometric and demographic indicators associated with high blood pressure in children aged 6 to 10 years in urban and rural areas of Minas Gerais.

**Method:**

this is a cross-sectional study with 335 children. Anthropometric, demographic and blood pressure data were collected. The statistics analyzes were performed using the chi-square, t-student, Mann-Whitney and logistic regression tests, and the odds ratio was the association measure.

**Results:**

the prevalence of high blood pressure was significantly higher among rural children. In the urban area, the chance of high blood pressure was higher in children who had a high body mass index (2.97 [1.13-7.67]) and in the rural area, in those who had increased waist circumference (35.4 [3.0-406.2]) and the age range of 9-10 years (4.29 [1.46-12.6]).

**Conclusion:**

elevated body mass index and waist circumference were important anthropometric indicators for high blood pressure, as well as age in children living in rural area. The evaluation of body mass index and waist circumference, in addition to nutritional assessments, represents an important action for the screening of high blood pressure in children from different territorial contexts.

## Abstract (pt)

**Objetivo:**

avaliar indicadores antropométricos e demográficos associados à pressão arterial elevada em crianças de 6 a 10 anos de idade de áreas urbana e rural de Minas Gerais.

**Método:**

estudo transversal realizado com 335 crianças. Foram coletados dados antropométricos, demográficos e de pressão arterial. As análises foram realizadas por meio dos testes Qui-quadrado, *t* student, Mann-Whitney e regressão logística, com cálculo do *odds ratio* como medida de associação.

**Resultados:**

a prevalência de pressão arterial elevada foi significativamente maior entre as crianças da área rural. Na área urbana, a chance de pressão arterial elevada foi maior nas crianças que possuíam o índice de massa corporal elevado (2,97 [1,13-7,67]) e, na área rural, naquelas que possuíam a circunferência da cintura aumentada (35,4 [3,0-406,2]) e faixa etária de 9-10 anos (4,29 [1,46-12,6]).

**Conclusão:**

o índice de massa corporal e a circunferência da cintura elevados foram importantes indicadores antropométricos para a pressão arterial elevada, assim como a idade em crianças residentes na área rural. A avaliação do índice de massa corporal e da circunferência da cintura, para além das avaliações nutricionais, representa importante ação para o rastreio de pressão arterial elevada em crianças de diferentes contextos territoriais.

## Abstract (es)

**Objetivo:**

evaluar los indicadores antropométricos y demográficos asociados a la presión arterial elevada de niños entre 6 y 10 años de edad en zonas urbana y rurale de Minas Gerais.

**Método:**

se trata de un estudio transversal realizado entre 335 niños. Se recopilaron datos antropométricos, demográficos y de presión arterial. Los análisis se realizaron con las pruebas de Chi-cuadrado, t de Student, Mann-Whitney y regresión logística, considerando el odds ratio como medida de asociación.

**Resultados:**

la prevalencia de la presión arterial elevada era significativamente más alta entre los niños de las zonas rurales. En la zona urbana, la probabilidad era mayor en los niños con índice alto de masa corporal [2,97(1,13-7,67)] y en la zona rural, en los que tenían más perímetro de cintura [35,4(3,0-406,2)] y grupo de edad de 9-10 años [4,29(1,46-12,6)].

**Conclusión:**

el índice de masa corporal y la circunferencia de la cintura altos fueron indicadores antropométricos importantes para la presión arterial elevada, así como la edad en niños residentes de la zona rural. La evaluación del índice de masa corporal y del perímetro de la cintura, además de las evaluaciones nutricionales, son factores importantes para el sondeo de la hipertensión arterial en niños de diferentes contextos territoriales.

## Introduction

Systemic arterial hypertension (SAH) is one of the most important global public health problems because it represents the leading cause of preventable death and the most common risk factor for cardiovascular diseases. In 2010, the global prevalence estimated of SAH in adult individuals was 31%, which was equivalent to 1.39 billion^(^ ^1^ ^)^. Data from the National Health Survey showed a prevalence of 21.4% in 2013 in Brazil^(^ ^2^ ^)^.

High blood pressure (BP) in children has reached increasing prevalence's in recent years^(^ ^3^ ^)^ and has increased, among other factors, due to the epidemic of childhood obesity that has been occurring in several countries^(^ ^4^ ^–^ ^5^ ^)^. There is evidence that children with high BP are at a significant risk for SAH in adulthood^(^ ^6^ ^–^ ^8^ ^)^.

In addition, children with elevated BP may present early complications such as coronary atherosclerosis and left ventricular hypertrophy, considered to be strong risk factors for early cardiac mortality^(^ ^9^ ^–^ ^10^ ^)^. Therefore, it is of great importance that these children are identified as early as possible so that adequate interventions provide control^(^ ^11^ ^)^.

Studies have shown that anthropometric indicators of adiposity can be used not only for nutritional assessments, but also to assess the risk of cardiovascular diseases, such as high blood pressure^(^ ^12^ ^–^ ^13^ ^)^. However, these studies do not consider the urban-rural residence condition^(^ ^14^ ^–^ ^17^ ^)^. In addition, high prevalence of high BP have been detected in children and adolescents in both urban and rural areas^(^ ^18^ ^–^ ^21^ ^)^. This demonstrates the importance of anthropometric assessments and alterations in BP in children from different territorial contexts.

Thus, this study aimed to evaluate anthropometric and demographic indicators associated with high blood pressure in children from 6 to 10 years of age in urban and rural areas of Minas Gerais.

## Method

This is a cross-sectional study developed in two public schools in the state of Minas Gerais, one in the Jardim Leblon neighborhood, in the northwest region of the capital Belo Horizonte, and the other in the rural district of São Pedro do Jequitinhonha, in the municipality of Jequitinhonha, in the northeast region of State.

In addition to population size, according to information from the United Nations Development Program (UNDP), the municipalities where the schools studied are located have different Human Development Index (HDI). Belo Horizonte has a total population of over 2.5 million inhabitants and an HDI of 0.810. The municipality of Jequitinhonha, has a total population estimated at just over 25 thousand inhabitants and an HDI of 0.615. São Pedro, located 43 km from the city hall, has a population of 1,600 inhabitants.

All children meeting the following criteria were included in the study: 6 to 10 years of age, being regularly enrolled in school, not using medications that could interfere with blood pressure, and being able to collaborate with blood collection procedures. The evaluations of the children were carried out between January and July 2015 in the school premises, in reserved rooms and by a team of previously trained nurses.

The rural school had 129 eligible children, but one was excluded because did not accept that blood pressure was measured. In the urban school, 210 children were eligible, but three were excluded, two of them were also not accepted for BP measurement and one for using medication to control BP.

Demographic information, such as sex and age, and anthropometric data, such as height, body weight and waist circumference, were collected from all participating children. Body weight and height were determined in a single measurement using a 0.1 kg precision digital scale and a 0.1 cm precision portable stadiometer (Alturexata^®^). The children were weighed barefoot and wearing light clothing. To get the height, the children stood without shoes, with heels firmly resting on the floor and their knees extended.

Weight and height measurements were used to calculate Body Mass Index (BMI) in kg/m^2^ using Anthro-Plus^®^ software (WHO, Geneva, Switzerland). BMI was classified as high when Z score was greater than +1^(^ ^22^ ^)^.

The waist circumference was evaluated twice with a non-elastic tape measuring the umbilical scar, with the child standing upright, with the abdomen naked and at the end of a normal expiration. For the final measure the average value of the two evaluations was used. Waist circumference was considered high in cases of percentile ≥ 90 for age and sex^(^ ^23^ ^)^. Height and waist circumference measures were used to calculate waist/height ratio, considered high when ≥ 0.5^(^ ^23^ ^)^.

Systolic blood pressure (SBP) and diastolic blood pressure (DBP) were assessed using a calibrated mercury sphygmomanometer after each child h-ad rested for at least 15 minutes. Blood pressure was measured three times, with an interval of five minutes, in the right arm and with an appropriate size cuff for the child's arm. The arm was placed on a table with the palm facing upwards and the cubital fossa at the level of the lower sternum.

The SBP was defined by the first Korotkoff sound and the DBP by the disappearance of the Korotkoff sound. High BP was defined as SBP and/or DBP of ≥90 percentile for age, sex and height^(^ ^11^ ^,^ ^24^ ^)^, considering the mean value of the three measures.

The collected data were inserted in double typing in Stata software version 12.1, in order to avoid transcription errors. This same software was used for descriptive and inferential statistical analysis. The evaluation of the normality of the independent variables (age, sex, height, weight, BMI, DBP, SBP, waist circumference and waist/height ratio) was done using the Shapiro-Wilk test. The dependent variable was elevated BP. The Student “t” and Mann-Whitney tests were used to evaluate the differences of means of the continuous variables between the two studied areas. Differences in the prevalence of high BP, elevated DBP and elevated SBP between the two areas were assessed by the Pearson Chi-square test.

Univariate logistic regression was performed and crude odds ratio (OR), with the respective 95% confidence intervals (95% CI), was estimated to identify the association between demographic and anthropometric characteristics with high BP in both areas. Subsequently, a logistic regression model was performed, estimating adjusted OR and 95% confidence intervals. The variables that presented p <0.20 in the univariate analysis, or those of theoretical importance described in the literature, were considered for the multivariate model.

In order to decide on the best fit for the multivariate model, the stepwise strategies were tested backward and forward. The Wald test was considered as a criterion for removing or adding variables to the model.

The Sperman correlation test was used to evaluate the presence of multicollinearity. Hosmer-Lesmeshow and Nagelkerke R2 tests were performed to evaluate the quality of fit of the final model. The level of statistical significance adopted was 5% (p≤0.05).

This study was approved by the Research Ethics Committee of the Federal University of Minas Gerais (No. 48087615.0.0000.5149). After authorization from the school directors, the free and informed consent of the parents and the free and informed consent of the children were obtained.

## Results

A total of 335 children participated in the study, of which 207 (61.8%) lived in the urban area and 128 (38.2%) in the rural area. The rural region concentrated a greater number of male children (56.3%) compared to urban (48.3%). The mean age of children living in rural area was higher than that of urban children (p <0.001). There was no statistically significant difference between the two groups in relation to weight, height, BMI, waist circumference and DBP. However, rural children had significantly higher SBP values than those in the urban area (p <0.001) and lower waist/height ratio (p = 0.022) (Table 1).

**Table 1:** Mean and standard deviations of age, anthropometric data and blood pressure measurements of children aged 6 to 10 years living in urban (Belo Horizonte) and rural (Jequitinhonha), MG, Brazil, 2015

	Urban (n=207)	Rural (n=128)	P-value
	Mean(SD*)	Mean(SD*)	
Age (years)	7.61 (1.38)	8.18(1.44)	**<0.001** †
Weight (kg)	27.82 (8.07)	27.49(6.67)	0.709‡
Height (cm)	128.31 (10.81)	128.54 (8.71)	0.469‡
BMI	16.64 (3.21)	16.42 (2.37)	0.704‡
Waist circunference§ (cm)	61.21 (9.09)	59.07 (6.44)	0.157‡
Waist-to-height ratio§	0.48 (0.07)	0.45 (0.04)	**0.022** ‡
Diastolic blood pressure	61.00 (8.24)	60.40 (9.04)	0.707‡
Sistolic blood pressure ‖	90.73 (10.66)	102.19 (11.74)	**<0.001** ‡

* Standard deviation;

† level of significance (*t test*);

‡ level of significance (*Mann-Whitney test*);

§ n urban = 181;

‖ mmHG.

**Figure 1:**
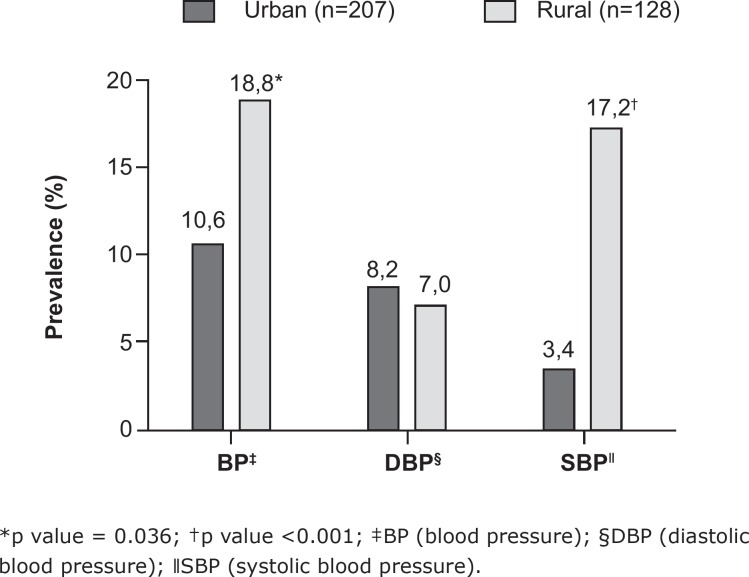
Prevalence of high blood pressure and high systolic and diastolic blood pressure in children aged 6 to 10 years living in urban (Belo Horizonte) and rural (Jequitinhonha), MG, Brazil, 2015

The overall prevalence of high BP was 13.7% in the children studied. In the rural area, the prevalence of high BP (18.8%) and high SBP (17.2%) was significantly higher in relation to the urban area (10.6% and 3.4%, respectively) (Figure 1).

Unadjusted analysis indicated that the prevalence of high BP in the urban area was significantly higher in children with a high BMI (OR 2.69, 95% CI 1.07-6.75). In the rural area, we observed that the prevalence of high BP was higher among children aged 9 to 10 years (OR 2.73, 95% CI: 1.07 – 6.94), with a high BMI (OR 4.10, 95% CI 1.54 – 10.09), with a high waist circumference (OR 14.70, 95% CI: 1.42 – 148.4), and with a waist/high height ratio (OR 4.60; 95% CI: 1.65-12.07).

After the adjusted analysis, in the rural area, age and waist circumference remained independently associated with the prevalence of elevated BP. In the urban area, children were more likely to present high BP when they had a high BMI (Table 2).

**Table 2:** Analysis of associations between demographic and anthropometric characteristics with high blood pressure among children aged 6 to 10 years living in urban (Belo Horizonte) and rural (Jequitinhonha), MG, Brazil, 2015

		Urban area (n=207)					Rural area(n=128)				
		Unadjusted-analysis			Adjusted-analysis*		Unadjusted-analysis			Adjusted-analysis†	
		%	OR‡ (95% CI)§	p‖	OR‡ (95% CI)§	p‖	%	OR‡ (95% CI)§	p‖	OR‡ (95% CI)§	p‖
**Gender**				0.867					0.254		
	Male	11,0	1.07 (0.44-2.06)				15.3	0.59 (0.24-1.45)		
	Female	10,3	1.00				23.3	1.00			
**Age**				0.772					**0.031**		**0.008**
	6-8	11,0	1.00				11.8	1.00		1.00	
	9-10	9,7	0.86 (0.32-3.32)				26.7	2.73 (1.07-6.94)		4.29 (1.46-12.6)	
**High BMI**				**0.031**		**0.027**			**0.033**		
	No	7.8	1.00		1.00		14.0	1.00			
	Yes	18.5	2.69 (1.07-6.75)		2.97 (1.13-7.67)		40.0	4.10 (1.54-10.9)			
**High waist circunference**					0.615				**0.021**		**0.004**
	No	10.6	1.00				16.9	1.00		1.00	
	Yes	14.3	1.40 (0.37-5.26)				75.0	14.70 (1.42-148.4)		35.4 (3.0-406.2)	
**High waist to height ratio**					0.940				**0.046**		
	No	10.9	1.00				14.0	1.00			
	Yes	11.3	1.04 (0.38-2.87)				42.9	4.60 (1.65-12.7)			

* Adjusted analysis (urban area) - Nagelkerke R^2^ = 0.051;

† Adjusted analysis (rural area) - Nagelkerke R^2^ = 0.250;

‡ OR = odds ratio;

§ IC = 95% confidence interval;

‖ p = Wald test value;

¶ BMI = Body Mass Index.

The variable “waist-to-height ratio” was not included in the final regression models due to the presence of collinearity with the variables waist circumference and elevated BMI. These variables were correlated by the Variance Inflation Factor (VIF) correlation test.

## Discussion

High waist circumference, central or abdominal fat index, as well as BMI, an indicator of body fat, were predictors of high BP in rural and urban children, respectively. The association between anthropometric indicators of overweight and high BP has also been mentioned in several national and international studies on childhood obesity^(^ ^16^ ^–^ ^17^ ^,^ ^19^ ^,^ ^25^ ^–^ ^30^ ^)^.

Recent changes in the eating patterns of Brazilian children, partly due to the improvement of general living conditions in both urban and rural areas, may be contributing to these associations^(^ ^31^ ^)^. It is believed that specific characteristics of the regions, such as economic, cultural and lifestyle differences, can mediate this association^(^ ^14^ ^)^.

In the rural area, the chance of older children aged 9 to 10 years old presenting with elevated BP was higher than in the age range from six to eight years. Although some studies also show a similar association^(^ ^17^ ^,^ ^19^ ^)^, it cannot be explained by the increase in age, since BP values were adjusted.

The general prevalence of high BP found in the studied children corroborates other studies developed with populations of the same age group^(^ ^15^ ^,^ ^27^ ^)^. In Brazil, this prevalence ranged from 3.8 to 40.6%^(^ ^14^ ^–^ ^15^ ^)^. However, the higher prevalence among rural children compared to urban children is a result that deserves to be highlighted. However, the scarcity of studies on the subject, considering both populations, makes comparisons difficult, indicating the need for greater attention to the urban/rural contrasts related to the health-disease process of children.

Blood pressure reading was recorded as the mean of three measurements performed on a single occasion. Therefore, the probability of occurrence of failures in the classification of children as high BP cannot be ruled out. Also, it is assumed as a limitation, the use of a North American reference to define high waist circumference, due to the absence of described patterns for Brazilian children.

As blood pressure measurement is not a routine practice in evaluating children^(^ ^32^ ^)^, high BMI and high waist circumference may indicate children with a higher chance of high BP. In the care of children with these high anthropometric indicators, the recommendation for the measurement of blood pressure should be more emphatic, considering the association of these indicators with elevated blood pressure levels. However, it is important to emphasize that elevated BP should not be confused with the diagnosis of systemic arterial hypertension. The latter, when in the pediatric population, can only be diagnosed in cases in which SBP and/or DBP remains greater than the reference for the 95th percentile in at least three different periods of measurements^(^ ^24^ ^)^.

Finally, it is important to include the evaluation of BMI and waist circumference as markers in the routine of evaluations of children, in schools and health units, as well as in medical and nursing consultations in urban and rural areas associated with high BP.

## Conclusion

In this study, anthropometric and demographic indicators were associated with high blood pressure in children from 6 to 10 years of age in urban and rural areas. The evaluation of BMI and waist circumference, in addition to nutritional assessments in children, represents an important action for the screening of high blood pressure in different territorial contexts.
